# Bis(acetato-κ*O*)(di-2-pyridyl­amine-κ^2^ *N* ^2^,*N* ^2′^)palladium(II)

**DOI:** 10.1107/S1600536812012093

**Published:** 2012-03-31

**Authors:** Kwang Ha

**Affiliations:** a School of Applied Chemical Engineering, The Research Institute of Catalysis, Chonnam National University, Gwangju 500-757, Republic of Korea

## Abstract

In the title complex, [Pd(CH_3_COO)_2_(C_10_H_9_N_3_)], the Pd^II^ ion is four-coordinated in a slightly distorted square-planar environment by two pyridine N atoms of the chelating di-2-pyridyl­amine (dpa) ligand and two O atoms from two anionic acetate ligands. The dpa ligand coordinates the Pd^II^ atom in a boat conformation of the resulting chelate ring; the dihedral angle between the pyridine rings is 39.3 (2)°. The two acetate anions coordinate the Pd^II^ atom as monodentate ligands and are located on the same sides of the PdN_2_O_2_ unit plane. The carboxyl­ate groups of the anionic ligands appear to be delocalized on the basis of the C—O bond lengths. Two complex mol­ecules are assembled through inter­molecular N—H⋯O hydrogen bonds, forming a dimer-type species. Inter­molecular C—H⋯O hydrogen bonds further stabilize the crystal structure.

## Related literature

For the crystal structures of the related Pd^II^ complexes [Pd*X*_2_(dpa)] (*X* = Cl or Br), see: Rauterkus *et al.* (2003 ▶); Yao *et al.* (2003 ▶).
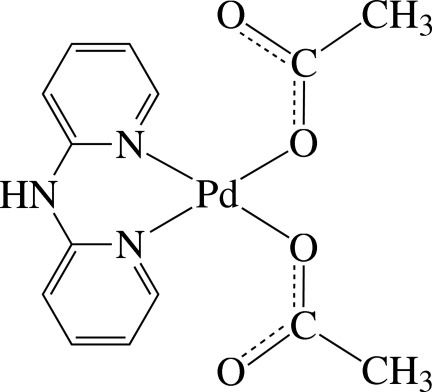


## Experimental

### 

#### Crystal data

[Pd(C_2_H_3_O_2_)_2_(C_10_H_9_N_3_)]
*M* *_r_* = 395.69
Monoclinic, 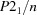

*a* = 8.565 (3) Å
*b* = 12.245 (5) Å
*c* = 14.230 (5) Å
β = 95.406 (8)°
*V* = 1485.8 (10) Å^3^
*Z* = 4
Mo *K*α radiation
μ = 1.27 mm^−1^
*T* = 200 K
0.24 × 0.10 × 0.10 mm

#### Data collection

Bruker SMART 1000 CCD diffractometer
Absorption correction: multi-scan (*SADABS*; Bruker, 2000 ▶) *T*_min_ = 0.777, *T*_max_ = 1.000
8823 measured reflections
2925 independent reflections
1807 reflections with *I* > 2σ(*I*)
*R*_int_ = 0.102

#### Refinement

*R*[*F* ^2^ > 2σ(*F* ^2^)] = 0.049
*wR*(*F* ^2^) = 0.116
*S* = 0.92
2925 reflections
205 parameters
H atoms treated by a mixture of independent and constrained refinement
Δρ_max_ = 0.99 e Å^−3^
Δρ_min_ = −0.81 e Å^−3^

### 

Data collection: *SMART* (Bruker, 2000 ▶); cell refinement: *SAINT* (Bruker, 2000 ▶); data reduction: *SAINT*; program(s) used to solve structure: *SHELXS97* (Sheldrick, 2008 ▶); program(s) used to refine structure: *SHELXL97* (Sheldrick, 2008 ▶); molecular graphics: *ORTEP-3* (Farrugia, 1997 ▶) and *PLATON* (Spek, 2009 ▶); software used to prepare material for publication: *SHELXL97*.

## Supplementary Material

Crystal structure: contains datablock(s) global. DOI: 10.1107/S1600536812012093/zq2158sup1.cif

Additional supplementary materials:  crystallographic information; 3D view; checkCIF report

## Figures and Tables

**Table:** 

Pd1—N3	2.003 (5)
Pd1—O1	2.004 (4)
Pd1—N1	2.004 (4)
Pd1—O3	2.006 (4)

**Table:** 

N3—Pd1—N1	89.13 (19)

**Table 2:** Hydrogen-bond geometry (Å, °)

*D*—H⋯*A*	*D*—H	H⋯*A*	*D*⋯*A*	*D*—H⋯*A*
N2—H2*N*⋯O2^i^	0.93 (6)	1.83 (7)	2.762 (7)	179 (6)
C2—H2⋯O4^ii^	0.95	2.58	3.302 (9)	133

Symmetry codes: (i) 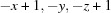; (ii) 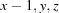.

**Fig. 1.**
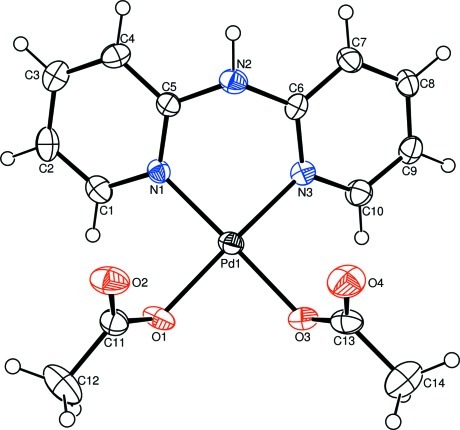
A structure detail of the title complex, with displacement ellipsoids drawn at the 40% probability level for non-H atoms.

**Fig. 2.**
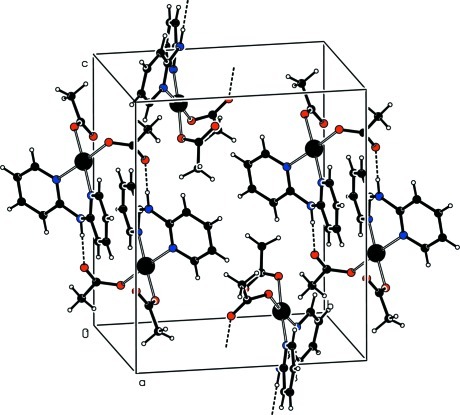
A view of the unit-cell contents of the title complex. Intermolecular N—H···O hydrogen-bond interactions are drawn with dashed lines.

## supplementary crystallographic information

### Comment

Crystal structures of Pd^II^ complexes with di-2-pyridylamine (dpa; C_10_H_9_N_3_) and halogen ions, [Pd*X*_2_(dpa)] (*X* = Cl or Br), have been reported previously (Rauterkus *et al.*, 2003; Yao *et al.*, 2003).

In the title complex, [Pd(C_2_H_3_O_2_)_2_(dpa)], the Pd^II^ ion is four-coordinated in a slightly distorted square-planar environment by two pyridine N atoms of the chelating dpa ligand and two O atoms from two anionic acetato ligands (Fig. 1). The dpa ligand coordinates the Pd atom in a boat conformation. The dihedral angle between the least-squares planes of the two pyridine rings is 39.3 (2)°. The Pd—N and Pd—O bond lengths are nearly equivalent [Pd—N: 2.003 (5) and 2.004 (5) Å; Pd—O: 2.004 (4) and 2.006 (4) Å] (Table 1). The two acetate anions coordinate the Pd atom as monodentate ligands *via* one O atom and are located on the same sides of the PdN_2_O_2_ unit plane. The carboxylate groups of the anionic ligands appear to be delocalized on the basis of the C—O bond lengths [C—O: 1.217 (7)–1.274 (8) Å]. Two complex molecules are assembled through intermolecular N—H···O hydrogen bonds, forming a dimer-type species (Fig. 2 and Table 2). Intra- and intermolecular C—H···O hydrogen bonds stabilize further the crystal structure (Table 2). The complex molecules are stacked into columns along the *a* axis. In the columns, several intermolecular π···π interactions between the pyridine rings are present, the shortest ring centroid···centroid distance being 4.646 (4) Å.

### Experimental

To a solution of Pd(CH_3_CO_2_)_2_ (0.1128 g, 0.502 mmol) in acetone (30 ml) was added di-2-pyridylamine (0.0873 g, 0.510 mmol) and stirred for 20 h at room temperature. After removal of the formed black precipitate by filtration, the solvent of the filtrate was evaporated, and the residue was washed with ether and dried under vacuum, to give a yellow powder (0.1462 g). Crystals suitable for X-ray analysis were obtained by slow evaporation from a CH_3_CN solution.

### Refinement

Carbon-bound H atoms were positioned geometrically and allowed to ride on their respective parent atoms: C—H = 0.95 Å (CH) or 0.98 Å (CH_3_) with *U*_iso_(H) = 1.2*U*_eq_(C) or 1.5*U*_eq_(methyl C). Nitrogen-bound H atom was located from the difference Fourier map and refined isotropically: N—H = 0.93 (6) Å. The highest peak (0.99 e Å^-3^) and the deepest hole (-0.81 e Å^-3^) in the difference Fourier map are located 0.87 Å and 0.83 Å from the Pd1 atom, respectively.

### Crystal data

**Table:**

[Pd(C_2_H_3_O_2_)_2_(C_10_H_9_N_3_)]	*F*(000) = 792
*M**_r_* = 395.69	*D*_x_ = 1.769 Mg m^−^^3^
Monoclinic, *P*2_1_/*n*	Mo *K*α radiation, λ = 0.71073 Å
Hall symbol: -P 2yn	Cell parameters from 2370 reflections
*a* = 8.565 (3) Å	θ = 2.2–25.4°
*b* = 12.245 (5) Å	µ = 1.27 mm^−^^1^
*c* = 14.230 (5) Å	*T* = 200 K
β = 95.406 (8)°	Block, yellow
*V* = 1485.8 (10) Å^3^	0.24 × 0.10 × 0.10 mm
*Z* = 4	

### Data collection

**Table:**

Bruker SMART 1000 CCD diffractometer	2925 independent reflections
Radiation source: fine-focus sealed tube	1807 reflections with *I* > 2σ(*I*)
Graphite monochromator	*R*_int_ = 0.102
φ and ω scans	θ_max_ = 26.1°, θ_min_ = 2.2°
Absorption correction: multi-scan (*SADABS*; Bruker, 2000)	*h* = −10→10
*T*_min_ = 0.777, *T*_max_ = 1.000	*k* = −11→15
8823 measured reflections	*l* = −17→17

### Refinement

**Table:**

Refinement on *F*^2^	Primary atom site location: structure-invariant direct methods
Least-squares matrix: full	Secondary atom site location: difference Fourier map
*R*[*F*^2^ > 2σ(*F*^2^)] = 0.049	Hydrogen site location: inferred from neighbouring sites
*wR*(*F*^2^) = 0.116	H atoms treated by a mixture of independent and constrained refinement
*S* = 0.92	*w* = 1/[σ^2^(*F*_o_^2^) + (0.0372*P*)^2^] where *P* = (*F*_o_^2^ + 2*F*_c_^2^)/3
2925 reflections	(Δ/σ)_max_ < 0.001
205 parameters	Δρ_max_ = 0.99 e Å^−^^3^
0 restraints	Δρ_min_ = −0.81 e Å^−^^3^

### Special details

**Table:**

Geometry. All e.s.d.'s (except the e.s.d. in the dihedral angle between two l.s. planes) are estimated using the full covariance matrix. The cell e.s.d.'s are taken into account individually in the estimation of e.s.d.'s in distances, angles and torsion angles; correlations between e.s.d.'s in cell parameters are only used when they are defined by crystal symmetry. An approximate (isotropic) treatment of cell e.s.d.'s is used for estimating e.s.d.'s involving l.s. planes.
Refinement. Refinement of *F*^2^ against ALL reflections. The weighted *R*-factor *wR* and goodness of fit *S* are based on *F*^2^, conventional *R*-factors *R* are based on *F*, with *F* set to zero for negative *F*^2^. The threshold expression of *F*^2^ > σ(*F*^2^) is used only for calculating *R*-factors(gt) *etc*. and is not relevant to the choice of reflections for refinement. *R*-factors based on *F*^2^ are statistically about twice as large as those based on *F*, and *R*- factors based on ALL data will be even larger.

### Fractional atomic coordinates and isotropic or equivalent isotropic displacement parameters (Å^2^)

**Table:**

	*x*	*y*	*z*	*U*_iso_*/*U*_eq_	
Pd1	0.51039 (5)	0.13264 (4)	0.30290 (3)	0.02916 (18)	
O1	0.3650 (5)	0.0500 (3)	0.2099 (3)	0.0364 (11)	
O2	0.3760 (6)	−0.1097 (4)	0.2847 (3)	0.0481 (13)	
O3	0.6539 (5)	0.1464 (4)	0.1999 (3)	0.0381 (11)	
O4	0.8275 (6)	0.0325 (4)	0.2707 (3)	0.0532 (14)	
N1	0.3580 (5)	0.1270 (4)	0.4014 (3)	0.0262 (11)	
N2	0.5642 (6)	0.1362 (5)	0.5225 (4)	0.0332 (13)	
H2N	0.584 (7)	0.126 (5)	0.587 (5)	0.044 (19)*	
N3	0.6529 (6)	0.2246 (4)	0.3902 (3)	0.0282 (12)	
C1	0.2031 (7)	0.1222 (5)	0.3758 (5)	0.0372 (16)	
H1	0.1674	0.1271	0.3107	0.045*	
C2	0.0939 (8)	0.1103 (5)	0.4403 (5)	0.0409 (17)	
H2	−0.0150	0.1079	0.4202	0.049*	
C3	0.1467 (8)	0.1020 (5)	0.5356 (5)	0.0390 (17)	
H3	0.0743	0.0927	0.5816	0.047*	
C4	0.3021 (7)	0.1074 (5)	0.5617 (4)	0.0359 (16)	
H4	0.3404	0.1006	0.6263	0.043*	
C5	0.4063 (7)	0.1232 (5)	0.4931 (4)	0.0263 (13)	
C6	0.6661 (7)	0.2077 (5)	0.4830 (4)	0.0262 (14)	
C7	0.7804 (7)	0.2595 (6)	0.5429 (5)	0.0364 (17)	
H7	0.7931	0.2425	0.6083	0.044*	
C8	0.8744 (8)	0.3357 (6)	0.5056 (5)	0.0386 (17)	
H8	0.9526	0.3732	0.5449	0.046*	
C9	0.8537 (7)	0.3569 (5)	0.4105 (4)	0.0353 (16)	
H9	0.9170	0.4101	0.3836	0.042*	
C10	0.7421 (8)	0.3015 (5)	0.3549 (5)	0.0368 (16)	
H10	0.7272	0.3179	0.2894	0.044*	
C11	0.3268 (7)	−0.0486 (6)	0.2199 (4)	0.0334 (16)	
C12	0.2142 (9)	−0.0922 (6)	0.1420 (6)	0.061 (2)	
H12A	0.2635	−0.1524	0.1103	0.092*	
H12B	0.1858	−0.0339	0.0964	0.092*	
H12C	0.1196	−0.1188	0.1684	0.092*	
C13	0.7840 (9)	0.0952 (6)	0.2073 (4)	0.0374 (17)	
C14	0.8867 (9)	0.1172 (7)	0.1291 (5)	0.060 (2)	
H14A	0.9949	0.1288	0.1561	0.089*	
H14B	0.8491	0.1827	0.0943	0.089*	
H14C	0.8827	0.0546	0.0861	0.089*	

### Atomic displacement parameters (Å^2^)

**Table:**

	*U*^11^	*U*^22^	*U*^33^	*U*^12^	*U*^13^	*U*^23^
Pd1	0.0331 (3)	0.0294 (3)	0.0243 (3)	−0.0019 (3)	−0.00082 (19)	−0.0001 (2)
O1	0.053 (3)	0.028 (3)	0.025 (2)	−0.004 (2)	−0.010 (2)	−0.002 (2)
O2	0.072 (4)	0.039 (3)	0.032 (2)	0.002 (3)	−0.004 (2)	0.005 (2)
O3	0.044 (3)	0.047 (3)	0.024 (2)	−0.001 (2)	0.004 (2)	0.000 (2)
O4	0.055 (3)	0.064 (4)	0.040 (3)	0.012 (3)	0.000 (2)	0.018 (3)
N1	0.026 (3)	0.025 (3)	0.027 (3)	0.000 (2)	−0.002 (2)	0.003 (2)
N2	0.037 (3)	0.033 (3)	0.029 (3)	0.002 (3)	−0.002 (2)	0.003 (3)
N3	0.031 (3)	0.024 (3)	0.029 (3)	0.002 (2)	0.003 (2)	0.002 (2)
C1	0.036 (4)	0.036 (4)	0.039 (4)	−0.001 (3)	−0.004 (3)	0.000 (3)
C2	0.032 (4)	0.030 (4)	0.061 (5)	−0.005 (3)	0.006 (3)	0.003 (4)
C3	0.038 (4)	0.035 (4)	0.046 (4)	0.002 (3)	0.013 (3)	0.004 (3)
C4	0.041 (4)	0.038 (4)	0.029 (3)	0.003 (3)	0.005 (3)	0.006 (3)
C5	0.031 (3)	0.019 (3)	0.029 (3)	−0.002 (3)	0.004 (3)	−0.006 (3)
C6	0.025 (3)	0.022 (4)	0.032 (3)	0.002 (3)	0.008 (3)	0.002 (3)
C7	0.028 (4)	0.049 (5)	0.032 (4)	0.002 (3)	−0.001 (3)	−0.006 (3)
C8	0.030 (4)	0.047 (5)	0.039 (4)	−0.010 (3)	0.002 (3)	−0.009 (3)
C9	0.034 (4)	0.032 (4)	0.042 (4)	−0.005 (3)	0.012 (3)	−0.004 (3)
C10	0.042 (4)	0.034 (4)	0.035 (4)	−0.004 (3)	0.004 (3)	0.003 (3)
C11	0.037 (4)	0.029 (4)	0.033 (4)	0.000 (3)	0.002 (3)	−0.002 (3)
C12	0.072 (6)	0.036 (4)	0.069 (5)	−0.008 (4)	−0.030 (5)	−0.002 (4)
C13	0.053 (5)	0.031 (4)	0.028 (4)	−0.003 (4)	0.004 (3)	−0.004 (3)
C14	0.064 (5)	0.062 (6)	0.056 (5)	0.010 (5)	0.024 (4)	0.012 (4)

### Geometric parameters (Å, º)

**Table:**

Pd1—N3	2.003 (5)	C3—H3	0.9500
Pd1—O1	2.004 (4)	C4—C5	1.396 (8)
Pd1—N1	2.004 (4)	C4—H4	0.9500
Pd1—O3	2.006 (4)	C6—C7	1.390 (8)
O1—C11	1.262 (7)	C7—C8	1.372 (9)
O2—C11	1.231 (7)	C7—H7	0.9500
O3—C13	1.274 (8)	C8—C9	1.372 (9)
O4—C13	1.217 (7)	C8—H8	0.9500
N1—C5	1.333 (7)	C9—C10	1.363 (9)
N1—C1	1.344 (7)	C9—H9	0.9500
N2—C5	1.386 (7)	C10—H10	0.9500
N2—C6	1.392 (8)	C11—C12	1.497 (9)
N2—H2N	0.93 (6)	C12—H12A	0.9800
N3—C6	1.331 (7)	C12—H12B	0.9800
N3—C10	1.341 (8)	C12—H12C	0.9800
C1—C2	1.378 (8)	C13—C14	1.506 (9)
C1—H1	0.9500	C14—H14A	0.9800
C2—C3	1.391 (9)	C14—H14B	0.9800
C2—H2	0.9500	C14—H14C	0.9800
C3—C4	1.350 (9)		
			
N3—Pd1—O1	175.98 (19)	N3—C6—N2	120.0 (6)
N3—Pd1—N1	89.13 (19)	C7—C6—N2	118.1 (5)
O1—Pd1—N1	92.25 (18)	C8—C7—C6	118.5 (6)
N3—Pd1—O3	91.49 (19)	C8—C7—H7	120.7
O1—Pd1—O3	86.87 (18)	C6—C7—H7	120.7
N1—Pd1—O3	176.08 (19)	C7—C8—C9	119.0 (6)
C11—O1—Pd1	124.0 (4)	C7—C8—H8	120.5
C13—O3—Pd1	119.4 (4)	C9—C8—H8	120.5
C5—N1—C1	118.1 (5)	C10—C9—C8	119.8 (6)
C5—N1—Pd1	121.6 (4)	C10—C9—H9	120.1
C1—N1—Pd1	120.2 (4)	C8—C9—H9	120.1
C5—N2—C6	125.6 (5)	N3—C10—C9	121.7 (6)
C5—N2—H2N	111 (4)	N3—C10—H10	119.2
C6—N2—H2N	115 (4)	C9—C10—H10	119.2
C6—N3—C10	118.9 (6)	O2—C11—O1	126.4 (6)
C6—N3—Pd1	121.2 (4)	O2—C11—C12	119.3 (6)
C10—N3—Pd1	119.9 (4)	O1—C11—C12	114.4 (6)
N1—C1—C2	122.6 (6)	C11—C12—H12A	109.5
N1—C1—H1	118.7	C11—C12—H12B	109.5
C2—C1—H1	118.7	H12A—C12—H12B	109.5
C1—C2—C3	118.5 (6)	C11—C12—H12C	109.5
C1—C2—H2	120.7	H12A—C12—H12C	109.5
C3—C2—H2	120.7	H12B—C12—H12C	109.5
C4—C3—C2	119.1 (6)	O4—C13—O3	125.1 (6)
C4—C3—H3	120.5	O4—C13—C14	120.1 (7)
C2—C3—H3	120.5	O3—C13—C14	114.7 (6)
C3—C4—C5	119.6 (6)	C13—C14—H14A	109.5
C3—C4—H4	120.2	C13—C14—H14B	109.5
C5—C4—H4	120.2	H14A—C14—H14B	109.5
N1—C5—N2	119.6 (5)	C13—C14—H14C	109.5
N1—C5—C4	122.0 (5)	H14A—C14—H14C	109.5
N2—C5—C4	118.4 (5)	H14B—C14—H14C	109.5
N3—C6—C7	121.8 (6)		
			
N1—Pd1—O1—C11	−66.8 (5)	C6—N2—C5—N1	38.0 (9)
O3—Pd1—O1—C11	117.1 (5)	C6—N2—C5—C4	−141.4 (6)
N3—Pd1—O3—C13	72.3 (5)	C3—C4—C5—N1	−3.8 (10)
O1—Pd1—O3—C13	−111.3 (5)	C3—C4—C5—N2	175.5 (6)
N3—Pd1—N1—C5	−35.4 (5)	C10—N3—C6—C7	−6.7 (9)
O1—Pd1—N1—C5	148.3 (5)	Pd1—N3—C6—C7	170.5 (5)
N3—Pd1—N1—C1	147.5 (5)	C10—N3—C6—N2	173.6 (6)
O1—Pd1—N1—C1	−28.7 (5)	Pd1—N3—C6—N2	−9.3 (8)
N1—Pd1—N3—C6	36.1 (5)	C5—N2—C6—N3	−37.1 (9)
O3—Pd1—N3—C6	−147.8 (5)	C5—N2—C6—C7	143.1 (6)
N1—Pd1—N3—C10	−146.8 (5)	N3—C6—C7—C8	4.6 (10)
O3—Pd1—N3—C10	29.3 (5)	N2—C6—C7—C8	−175.6 (6)
C5—N1—C1—C2	−1.9 (10)	C6—C7—C8—C9	−0.8 (10)
Pd1—N1—C1—C2	175.2 (5)	C7—C8—C9—C10	−0.7 (10)
N1—C1—C2—C3	−0.6 (10)	C6—N3—C10—C9	5.0 (9)
C1—C2—C3—C4	1.0 (10)	Pd1—N3—C10—C9	−172.2 (5)
C2—C3—C4—C5	1.1 (10)	C8—C9—C10—N3	−1.3 (10)
C1—N1—C5—N2	−175.2 (5)	Pd1—O1—C11—O2	−3.0 (10)
Pd1—N1—C5—N2	7.7 (8)	Pd1—O1—C11—C12	178.9 (5)
C1—N1—C5—C4	4.1 (9)	Pd1—O3—C13—O4	3.9 (9)
Pd1—N1—C5—C4	−173.0 (5)	Pd1—O3—C13—C14	−176.2 (5)

### Hydrogen-bond geometry (Å, º)

**Table:**

*D*—H···*A*	*D*—H	H···*A*	*D*···*A*	*D*—H···*A*
N2—H2*N*···O2^i^	0.93 (6)	1.83 (7)	2.762 (7)	179 (6)
C1—H1···O1	0.95	2.50	2.983 (8)	111
C2—H2···O4^ii^	0.95	2.58	3.302 (9)	133
C10—H10···O3	0.95	2.51	2.954 (8)	109

Symmetry codes: (i) −*x*+1, −*y*, −*z*+1; (ii) *x*−1, *y*, *z*.
